# The role of the anterior cingulate cortex in anxiety control, from circuits to disorders

**DOI:** 10.3389/fpsyt.2026.1719721

**Published:** 2026-08-26

**Authors:** Emily Dew, Alexis Jones, Shalini Saggu, Lucas Pozzo-Miller, Qin Wang

**Affiliations:** 1 Department of Neuroscience and Regenerative Medicine, Medical College of Georgia at Augusta University, Augusta, GA, United States; 2 Department of Pediatrics & Human Development, College of Human Medicine, Michigan State University, Grand Rapids, MI, United States

**Keywords:** anterior cingulate cortex, anxiety, anxiety disorders, circuits, cytoarchitecture

## Abstract

Anxiety is an adaptive response essential for survival that integrates external stimuli, internal states, memory, and decision making to respond to perceived threats. When dysregulated, it can become excessive and persistent, contributing to psychiatric and neurological disorders. The anterior cingulate cortex (ACC) integrates cognitive, emotional, interoceptive, and autonomic information and serves as a key hub in anxiety-related circuits. However, its subregion- and pathway-specific roles remain incompletely integrated across species. This review examines the cytoarchitectural and cellular organization of ACC subregions and their proposed correspondence in humans, non-human primates, and rodents. We then review ACC connectivity with the amygdala, hippocampus, insula, and bed nucleus of the stria terminalis and propose an integrative model in which ACC effects depend on subregion, projection target, and behavioral context. We also summarize structural, functional, connectivity, and neurochemical alterations of the ACC in anxiety-related disorders and neurodegenerative diseases with prominent anxiety symptoms. Finally, we discuss the strengths and limitations of rodent and non-human-primate models and identify priorities for future mechanistic and translational studies. Together, this review provides a timely and comprehensive view of the ACC’s role in adaptive and pathological anxiety and offers a framework for advancing mechanistic and translational research.

## Introduction

Anxiety is an adaptive response that integrates external stimuli, memory, internal state, and decision making to react to a threat or environmental challenges and is necessary for survival. It is accompanied by autonomic and somatic changes, including increased heart rate, blood pressure, respiratory activity, pupil dilation, and muscle tension, together with heightened vigilance and altered attention (1–3). Many of these physiological and behavioral changes also occur during fear. Despite the close relationship and partial overlap between fear and anxiety, these two phenomena can be distinguished at the conceptual, behavioral, and circuit levels. Fear is generally a phasic reaction to an immediate and clearly defined/identifiable threat, whereas anxiety is a more sustained and anticipatory response to uncertain, distant, or potential threats (3–7). Crucially, compared with phasic fear, anxiety depends more strongly on distributed cortical and limbic circuitry involved in threat anticipation, sustained monitoring, and the processing of uncertainty (3, 4, 6, 7). Many core neural mechanisms underlying threat detection, defensive responses, and autonomic regulation are broadly conserved across mammals, providing an important foundation for cross-species investigation of fear and anxiety (1, 8, 9).

While anxiety is a biological response that aids decision making and promotes survival, it can become pathological when it is excessive, persistent, disproportionate to the actual threat, or sufficiently severe to impair daily function. Pathological anxiety is both a disorder itself and a common symptom or comorbidity in other neurological and psychiatric conditions. Anxiety disorders mainly include generalized anxiety disorder, social anxiety disorder, and panic disorder (2, 10). Patient-reported symptoms and physician-observable signs are both somatic and psychological, including excessive worry, restlessness, fatigue, irritability, difficulty concentrating, sleep disturbances, muscle tension, and avoidance behavior (2, 3, 8, 11). In addition to these signs and symptoms, panic disorder involves panic attacks, which are sudden bursts of severe anxiety accompanied by cardiac, respiratory, neurological, gastrointestinal, and autonomic arousal (11, 12). Obsessive-compulsive disorder and post-traumatic stress disorder were previously also classified as anxiety disorders and both exhibit pathological anxiety as a major symptom (10). Common comorbidities of an anxiety disorder include other anxiety disorders, depression, bipolar disorders, personality disorders, and substance abuse disorders (12). Pathological anxiety is also present in Alzheimer’s Disease (AD), Parkinson’s Disease (PD), Huntington’s Disease, amyotrophic lateral sclerosis, lupus, and prion diseases (13–18).

The anxiety circuitry detects, evaluates, and processes uncertain or potentially threatening stimuli and coordinates anticipatory, cognitive, autonomic, and behavioral responses (3, 4, 6). Anxiety-related brain networks involve interacting regions, including the anterior cingulate cortex (ACC), amygdala, insula, hippocampus, bed nucleus of the stria terminalis (BNST), hypothalamus, and periaqueductal gray (1). Within these networks, the ACC integrates emotional, cognitive, interoceptive, and autonomic information and contributes to adaptive behavioral responses during uncertainty, conflict, and threat (19–21). A deeper understanding of how the ACC mediates these regulatory functions could provide important insights into the biological basis of anxiety and the mechanisms disrupted in its pathological forms. Despite its importance, however, the specific anatomical, cellular, and circuit-level contributions of the ACC to adaptive and pathological anxiety have not yet been comprehensively integrated.

In this review, we describe the anatomical and cellular organization of the ACC, examine its interactions with key anxiety-related regions, and summarize ACC alterations in anxiety disorders and neurological diseases with prominent anxiety symptoms. Since rodent models are increasingly used for mechanistic analyses of anxiety, we also evaluate primate and rodent models, emphasizing both conserved circuit motifs and important species differences. Through this synthesis of literature, our goal is to provide an integrated view of ACC’s role in adaptive and pathological anxiety and to identify priorities for future mechanistic and translational studies.

## ACC anatomy and cellular features in humans and non-human primates

**Figure 1:**
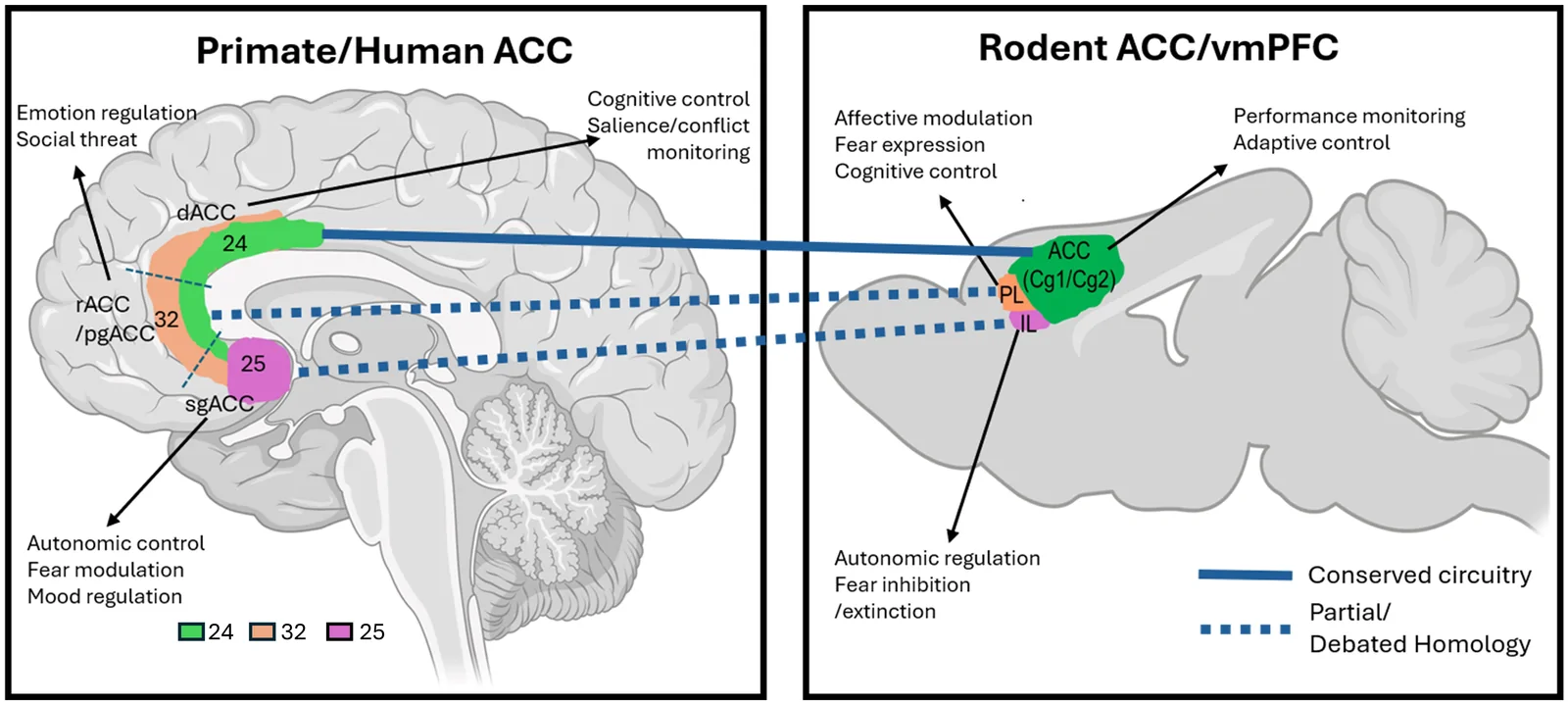
Cross-species organization of ACC subregions. Schematic representation of human and non-human-primate ACC subdivisions, including the dorsal ACC (dACC), rostral/pregenual ACC (rACC/pgACC), and subgenual ACC (sgACC), and their proposed correspondences to rodent medial frontal regions, including the ACC (Cg1/Cg2), prelimbic cortex (PL), and infralimbic cortex (IL). BA24/32 indicates that the functionally defined dACC spans dorsal portions of Brodmann area 24 and adjacent area 32. Solid connectors indicate relatively conserved anatomical or circuit relationships, whereas dashed connectors indicate partial or working correspondences rather than strict one-to-one homology. The schematic emphasizes cross-species functional and circuit-level correspondence.

In humans and non-human primates, the ACC occupies the frontal third of the cingulate cortex and is commonly described in three partially overlapping subregions along the rostrocaudal and dorsoventral axis, the dorsal ACC (dACC), rostral/pregenual ACC (rACC/pgACC), and subgenual ACC (sgACC). The dACC encompasses dorsal portions of Brodmann area 24 and adjacent area 32, the rACC/pgACC consists of the rostral portions of areas 24 and 32, and the sgACC is the ventral-most part of the ACC centered on area 25 with extension into adjacent ventromedial portions of area 24 and 32 (21–25) (**Figure 1**, left). The exact boundaries and terminology used for these subregions vary somewhat across anatomical and imaging studies. The ACC differs from the posterior cingulate cortex (PCC) and midcingulate cortex (MCC) in both cytoarchitecture and function. The ACC is mainly agranular or dysgranular and has less distinct cortical lamination than the PCC (23, 25). It also contains a concentrated population of von Economo neurons (VEN) in layer V and spindle-shaped neurons (25–27). By contrast, PCC layers are well differentiated and richly laminated (23, 28). The MCC sits between the ACC and PCC and has neurofilament-expressing neurons in layer IIIc and large layer Vb pyramidal neurons that distinguish it from the ACC (29, 30). The ACC and MCC also differ in their patterns of connectivity. The ACC, particularly its rostral/pregenual and subgenual regions, is strongly interconnected with the amygdala and other limbic and autonomic structures, whereas the MCC is more strongly connected with parietal and motor networks (23, 24, 29, 30). The MCC also contains cingulate motor areas involved in action selection and the planning and execution of movement (30). Functionally, the ACC contributes to emotional and autonomic regulation and cognitive-affective integration, the PCC to internally directed, evaluative, and memory-related processing, and the MCC to action selection and skeletomotor control (19, 21, 23, 30, 31).

The cerebral cortex is typically organized into six layers, each distinguished by its characteristic cell types and connectivity. However, the laminar organization of the ACC differs across its cytoarchitectonic areas. Areas 24 and 25 are agranular and lack a distinct layer IV, whereas area 32 is dysgranular and contains a thin or incompletely developed layer IV (28, 32, 33). Layer I (the molecular layer) consists primarily of long-range axon projections and the apical dendrites of pyramidal cells from the other ACC layers, with sparse GABA interneurons. This organization allows layer I to integrate inputs from different cortical and subcortical sources and regulate pyramidal neuron activity (25, 33). Layer II is the external granular layer, composed of small, closely packed cells, and layer III is the external pyramidal layer, which is composed of medium pyramidal neurons. In the sgACC, layer II can be divided into a neuron-dense layer IIa and a relatively neuron-sparse layer IIb, while layer III is comparatively thin (28). Layers II/III receive input from layer I and send output to layer V/VI and are also involved in other corticocortical communications (28, 32). Layer V is the internal pyramidal layer, and layer VI is the polymorphic layer. These pyramidal neurons receive and process input from layer II/III and contribute to long-range cortical, subcortical, and thalamic projections (28, 32).

Interneurons and non-neuronal cells also show layer-related distributions. Human ACC transcriptomic datasets identify VIP-, LAMP5-, and RELN-expressing interneuron populations mainly in superficial layers and SST- and TAC1-expressing populations more prominently in deeper layers (34, 35). These molecular classes are heterogeneous and should not be assumed to have identical circuit functions. Astrocytes, oligodendrocytes, microglia, and oligodendrocyte precursor cells are found across the cortical layers (34, 35). A summary of the major laminar cell types and interconnections is provided in **Table 1**.

**Table 1:** Cellular features of the ACC.

Layer	Composition	Afferent projection	Efferent projection	Cell types
I	Molecular layer (neuropil with low soma density)	Long-range corticocortical and thalamic axons	Mostly intracortical (local modulation)	Sparse interneurons
II/III	External pyramidal	Layer I + local and corticocortical inputs	Layer V/VI and corticocortical outputs	Small/medium pyramidal neurons; interneurons
V	Internal pyramidal	Layer II/III + local circuits	Outputs to subcortical and some cortical regions	Large pyramidal neurons; interneurons; VENs
VI	Polymorphic	Layer II/III + local circuits	Corticothalamic and corticocortical outputs	Pyramidal neurons; interneurons

Recent single-nucleus RNA sequencing of human samples has revealed molecular features and heterogeneities of different cell populations in the ACC (34, 35). For example, interneuron clusters are divided into VIP, SST, TAC1, LAMP5, and RELN interneuron subclasses based on their specific expression of those genes (34, 35). The VIP, LAMP5, and RELN interneurons are classified as disinhibitory because they inhibit other interneurons (34), although LAMP5- and RELN-expressing populations can also provide direct inhibition of pyramidal neuron dendrites. SST and TAC1 interneurons share the same origin from the medial ganglionic eminence (MGE) and are grouped as MGE interneurons (34). Computational integration of ACC cell-type signatures with disease-associated gene sets predicted that interneurons may be particularly vulnerable to neurological and psychiatric disorders (34). In particular, SST- and VIP-expressing populations show some of the strongest disease-gene enrichment (34). While altered disinhibitory interneurons are associated with disorders involving cognition and behavior, seizure, and PD, altered MGE interneurons are associated with addiction, schizoaffective disorder, neurogenic inflammation, and generalized hyperkinesia (34). Similarly, pyramidal and non-neuronal populations also showed subtype-specific patterns of disease association (34). Within the sgACC, laser-capture RNA sequencing found stronger transcriptional differences between layers III and V than among the cytoarchitectonic areas sampled within the sgACC. Layer III was enriched for pathways related to cell junctions and dendritic spines, whereas layer V was enriched for pathways related to presynaptic function and brain development (39). However, currently available transcriptomic datasets are concentrated largely in the sgACC, and subregion-specific data remain limited, preventing direct comparisons among the dACC, pgACC, and sgACC. Further studies directly comparing these subregions will be needed to determine how their cellular and molecular differences contribute to distinct functions in anxiety.

The ACC and insula have a distinct population of projection neurons known as von Economo neurons, characterized by a spindle-shaped soma with a single thick apical dendrite and a single thick basal dendrite (25–27). VENs are generally considered excitatory (glutamatergic) neurons distributed primarily in layer V of the ACC and insula (27, 36). Transcriptomic analyses indicate that VENs express a molecular profile that differs from neighboring layer III and layer V pyramidal neurons (37). Because of their large size and long-range projection morphology, VENs are hypothesized to support rapid long-range signal transmission (25, 27). Their potential high synaptic connectivity in ACC-insula communication remains to be established experimentally (19). Although initially thought to exist only in humans, apes, and macaque monkeys, they have also been reported in other large-brained mammals (27, 38–43). Based on their distribution in the ACC and anterior insula, VENs have been proposed to contribute to salience processing, autonomic regulation, and rapid responses to socially relevant information (25, 38, 42), but these functions have not been directly established. Microdissected-cell RNA sequencing of layer V VENs found 344 differentially expressed genes when compared to layer III and layer V pyramidal neurons (36). Among the altered genes, the upregulated ones in VENs are associated with social, emotional, and autonomic functions (36), suggesting a molecular signature consistent with proposed roles for VENs. Alterations in VENs have been reported in autism, schizophrenia, frontotemporal dementia, and AD (43–47), all of which are characterized by social and emotional deficits. Their specific functional role in social behavior remains an active area of investigation.

A molecularly, physiologically, and connectionally equivalent VEN population has not been established in rodents, although neurons with some VEN-like morphological features have been described (42). This limits direct cross-species comparison at the level of this specialized cell type and supports greater emphasis on conserved circuit motifs and connectivity principles in translational studies.

## Cytoarchitectural and functional homology of ACC between rodents and primates

Rodents are valuable experimental systems for validating the role of the ACC in anxiety and investigating the underlying cellular and circuit mechanisms. However, interpretation of rodent findings requires careful comparison of the structure and function of rodent and primate medial frontal regions. Although rodent and primate brains differ substantially in their overall organization, the rodent ACC shares conserved cytoarchitectural features and some similarities in intrinsic functional organization with its primate counterpart. As in primates, the rodent ACC can be distinguished from the MCC by differences in cytoarchitecture and connectivity, including comparatively stronger retrosplenial and parietal connections of the MCC (48–50). Other broadly conserved features include agranular or dysgranular cytoarchitecture and extensive connections with cortical, limbic, thalamic, hypothalamic, and brainstem regions involved in emotional, autonomic, and behavioral regulation (32, 48–53). At the same time, large-scale connectivity also shows important species differences (48, 51).

The rodent ACC is often considered within the broader medial prefrontal cortex (mPFC) framework. The rodent mPFC includes cytoarchitectonically and connectionally distinct regions, commonly including the ACC, prelimbic cortex (PL), and infralimbic cortex (IL), although the terminology and boundaries vary across atlases and studies (9, 49, 52, 54). Rodent area 24 is generally compared with primate area 24 and dorsal cingulate territories, whereas PL and IL are sometimes compared with primate pgACC/rACC and sgACC/vmPFC regions, respectively (9, 32, 48, 51, 55, 56). However, these comparisons depend on the anatomical, connectivity, or functional criteria being considered and should be regarded as working correspondences rather than strict one-to-one homologies (**Figure 1**). Importantly, these proposed correspondences do not imply equivalent functions. In particular, primate areas 25 and 32 can exert effects on threat-related behavior that differ from those commonly attributed to rodent IL and PL, respectively (57). Macaque area 25 projections preferentially engage excitatory and potentially disinhibitory circuits within the basal amygdala rather than reproducing the rodent IL projection to inhibitory intercalated cells (58, 59). Thus, the sign and behavioral function of a primate ACC pathway should not be inferred directly from its proposed rodent homologue.

In terms of connectivity, a default-mode-like network first described in primates has also been identified in awake rodents using fMRI studies (60). More broadly, structural and functional connections between medial frontal regions and areas involved in emotion and cognition are partly conserved across rodents and primates, although their relative organization differs across species (9, 48, 51). Anterograde tracing studies in mice show that ACC projections are predominantly ipsilateral, with more limited contralateral projections. Major targets include cortical association and sensory regions, the striatum, amygdala, thalamus, hypothalamus, periaqueductal gray, and other brainstem regions (50, 53). The rodent ACC also receives inputs from cortical regions, the hippocampus, amygdala, thalamus, hypothalamus, and brainstem neuromodulatory systems (49, 52). Functionally, the rodent mPFC/ACC regions show partial parallels with primate medial frontal regions in regulating fear, stress, and other emotional responses (9, 19). fMRI comparisons between high- and low-anxiety rat strains report differences in mPFC/ACC intrinsic connectivity, and lesions within this area can reduce anxiety-like behavior and alter the response to anxiogenic stimuli (61–63). More direct ACC studies show that selective inhibition of neurabin within the ACC reduces anxiety-like behavior in mice, while Fos-mapping studies implicate ACC recruitment in the behavioral effects of benzodiazepines during repeated elevated-plus-maze exposure in rats (64, 65). The rodent mPFC/ACC is also implicated in social behaviors. For example, mPFC/ACC lesions in rats reduce social behavior and impair aspects of social memory (66).

Nonetheless, there are notable species differences in the ACC organization. Rodents show less pronounced rostrocaudal and dorsoventral differentiation of cingulate and medial frontal regions than primates. They also lack an established molecularly, physiologically, and connectionally equivalent population of the VENs found in the primate ACC, as discussed above. Rodent models should therefore be interpreted as capturing conserved circuit mechanisms involved in threat evaluation, avoidance, arousal, and emotional regulation rather than reproducing the full organization and functional repertoire of the human ACC.

Despite these limitations, rodents remain valuable models for studying ACC-linked mechanisms relevant to anxiety. They permit precise genetic, molecular, cell type-specific, and projection-specific manipulations that are difficult to perform in primates, as well as longitudinal studies across development, aging, and disease progression. Although rodent behavioral paradigms do not reproduce human anxiety disorders in their entirety, they provide measurable readouts of conserved behavioral dimensions. Common assays include the open-field, light–dark box, elevated plus or zero maze, and novelty-suppressed feeding tests (67–69). Measures such as avoidance of exposed areas, risk assessment, and behavioral inhibition are sensitive to anxiolytic and anxiogenic manipulations and can be used to examine anticipatory threat and anxiety-like states. Fear-conditioning and extinction paradigms assess threat learning, generalization, and safety signaling (4, 6, 7), whereas stress-reactivity and social-interaction paradigms measure hyperarousal, social avoidance, and susceptibility or resilience to repeated stress (70). Using multiple assays to obtain complementary behavioral and physiological measures reduces reliance on any single test as a model of anxiety. In the context of ACC research, these assays are most informative when combined with subregion-, cell type-, or projection-specific manipulations that establish how defined ACC circuits regulate particular dimensions of anxiety-related behavior.

## ACC connectivity and circuits in anxiety

In this section, we primarily review the literature from human and non-human primates, with selective inclusion of rodent studies that provide mechanistic evidence or illustrate conserved circuit motifs. From an integrative perspective, the contribution of the ACC to anxiety arises through its interactions with the amygdala, hippocampus, insula, BNST, and other cortical and subcortical regions. Although many basic circuit motifs are conserved across mammals, the anatomical correspondence between individual rodent and primate medial frontal regions is not always direct (8, 9, 51, 57). Rodent findings are therefore identified explicitly below.

### Subregion-specific connectivity of the ACC

Different subregions of the ACC have distinct intrinsic functional connectivity profiles as demonstrated by resting-state fMRI and supported by anatomical tract-tracing studies (21, 22, 31, 71) (**Table 2**). Terminology should be interpreted carefully because much of the region labeled as dACC in human imaging studies overlaps with the anterior midcingulate cortex (aMCC) in cytoarchitectonic classifications (9, 21, 23). We retain the terminology used by the original studies but use dACC/aMCC when referring more broadly to this region.

**Table 2:** Connectivity and function of ACC subregions in humans and non-human primates.

ACC subregion	Strong connectivity	Main functional associations	Key refs
dACC	Anterior insula; thalamus; dorsal/lateral PFC; parietal cortex; dorsal striatum; premotor/motor networks (in primates)	Salience detection; performance monitoring; goal-directed control; motor planning/selection and modulation of descending motor pathways (via cingulate motor territories)	(21–23, 31, 71)
rACC/ pgACC	Other ACC subregions; amygdala; medial frontal/frontopolar cortex; orbitofrontal cortex	Emotional appraisal/regulation; context-dependent autonomic integration; affective components of pain; modulation of amygdala responses during emotional conflict processing	(20, 24, 72, 75)
sgACC	Hypothalamus; nucleus accumbens/ventral striatum; amygdala; BNST; hippocampus; periaqueductal gray	Visceromotor/autonomic control; visceral integration; affective learning; sustained mood-related states	(23, 58, 59, 73–75, 78, 79)

The dACC/aMCC has strong connections to brain regions involved in salience, performance monitoring, action selection, and cognitive control, including the anterior insula, thalamus, dorsal/lateral prefrontal and parietal cortices, and dorsal striatum (21, 22, 71–73). This is consistent with its roles in salience detection, performance monitoring, and goal-directed control. In addition, dACC connections with the premotor/motor networks may contribute to the modulation of descending motor pathways in primates (22, 23). The rACC/pgACC has stronger connectivity with other ACC subregions, the amygdala, medial frontal/frontopolar and orbitofrontal cortices, consistent with emotional appraisal/regulation, context-dependent autonomic integration, and affective components of pain (20, 24, 72, 74, 75). The sgACC, particularly area 25, is connected with the amygdala, anterior hippocampus, hypothalamus, BNST, ventral striatum, periaqueductal gray, and interoceptive cortical regions. These connections place the sgACC in a position to influence sustained affective states, autonomic activity, motivation, and responses to uncertain threat (58, 59, 73–75). Together, these connectivity patterns offer a circuit-level framework for understanding the role of ACC in regulating emotion, cognition, and autonomic functions. However, connectivity alone does not establish whether a given ACC pathway suppresses or facilitates anxiety-related responses.

### ACC-amygdala circuits

The amygdala is a major component of brain networks involved in both fear and anxiety. The basolateral amygdala (BLA) complex integrates sensory, contextual, and value-related information, whereas the central amygdala (CeA) and related extended-amygdala structures organize autonomic, endocrine, and defensive responses (4, 7, 76, 77). Although these functions are traditionally investigated in fear paradigms, they are also involved in processing sustained and uncertain threat and are particularly relevant to the ACC circuitry. The ACC and amygdala are strongly and bidirectionally connected, but the strength, direction, and functional effects of these interactions vary across ACC subregions, amygdala nuclei, and behavioral conditions (58, 59, 73, 75).

Primate tracing studies demonstrate prominent projections from the ACC to the amygdala. sgACC projections are broadly distributed across the main amygdala nuclei, whereas pgACC projections are more restricted and nested within sgACC terminal fields. The two pathways converge on common putative excitatory neurons in regions of the basal and accessory basal nuclei (59). In addition, ACC output can engage both excitatory and inhibitory amygdala microcircuits (58). Reciprocal projections from the amygdala also differ across ACC subregions. Amygdala inputs to the dACC and pgACC arise predominantly from the basal nucleus, whereas the sgACC receives a greater proportion of input from the accessory basal nucleus and some input from the lateral nucleus (75). Causal studies in marmosets further demonstrate that ACC subregions have different effects on threat-related behavior. Inactivation of area 25 reduces behavioral and cardiovascular responses to anticipated threat, whereas inactivation of area 32 impairs threat discrimination and promotes generalization (57). Conversely, overactivation of area 25 increases behavioral, cardiovascular, endocrine, and amygdala responses to threat (78). Selective activation of the area 25–amygdala pathway also increases responses to uncertain threat, directly showing that this pathway can facilitate rather than suppress anxiety-related behavior (79).

Rodent studies provide additional circuit-level evidence, although most have examined conditioned or innate fear rather than anxiety itself. In mice, there are monosynaptic, glutamatergic projections from the ACC/mPFC regions to the BLA, supporting generalized contextual fear. Inhibiting these projections reduces freezing in a novel, nonthreatening context without reducing fear in the original conditioning context, suggesting that the ACC–BLA pathway contributes to fear generalization (80). In a naturalistic predator-threat model, the anterior cingulate area contributes to both the acquisition and expression of contextual fear. Its projection to the BLA contributes to fear-memory acquisition, whereas its projection to the dorsolateral periaqueductal gray contributes to the expression of contextual defensive responses (81). Taken together, evidence from primates and rodents supports subregion-, pathway-, and context-dependent roles for ACC-amygdala circuits in threat processing.

### ACC-hippocampal circuits

The hippocampus (HPC) regulates contextual and episodic memory. Its interaction with the ACC supports threat evaluation, emotional responses, and decisions under uncertainty (82–84). In humans, the anterior hippocampus is recruited during approach–avoidance conflict and increasing threat, supporting its role in anxiety-relevant behavioral inhibition and avoidance (85). In non-human primates, injections of fluorescently-labeled tracers into the anterior HPC demonstrate direct projections to ACC areas 24a, 25, and 32, with the densest terminations in posterior area 25 (74). These projections provide an anatomical pathway through which current context and past experience may influence ACC-dependent emotional, autonomic, and goal-directed processing.

Tracing studies from rodents demonstrate robust direct projections from the HPC to mPFC/ACC regions (86–89), while direct return projections are generally not observed (86). Rodent studies also provide stronger mechanistic evidence for hippocampal–medial frontal interactions in anxiety-like behavior. When mice enter anxiogenic environments, activity in the ventral hippocampus and mPFC becomes more synchronized at theta frequencies, and stronger synchrony is associated with greater avoidance (90). Inhibiting ventral hippocampal inputs to the mPFC reduces avoidance and alters prefrontal representations of threatening locations, whereas theta-frequency stimulation of the same pathway increases avoidance (91, 92). However, this pathway is not uniformly anxiogenic, as different hippocampal projections can promote either approach or avoidance through distinct prefrontal microcircuits (93). Anatomically, the connection is mainly directed from the hippocampus to the mPFC, while communication in the opposite direction occurs largely through intermediary structures, particularly the thalamic nucleus reuniens (88, 94, 95). Taken together, the anterior hippocampus in primates and ventral hippocampus in rodents provide contextual, memory-related, and uncertainty-related information to medial frontal regions. The current findings also highlight the need for direct functional tests of the ACC-HPC circuits in primates.

### ACC-insula circuits

The insula is a heterogeneous region that links internal bodily states with emotional, cognitive, and behavioral processes and contributes to emotion recognition and processing, social motivation, homeostatic regulation, and reward (96, 97). In humans, the anterior insula is functionally connected with the pgACC and aMCC, whereas the middle and posterior insula show stronger connectivity with more posterior portions of the MCC (97). Together, the anterior insula and dACC/aMCC form major cortical nodes of the salience network, which identifies behaviorally relevant internal and external events and helps coordinate cognitive, autonomic, and behavioral responses (71). The ACC and anterior insula are also jointly recruited during interoceptive and anxiety-related processing. Activity in both regions increases during attention to internal bodily signals and overlapping areas of the anterior insula and ACC are activated during interoceptive awareness and phobic symptom provocation (98, 99). In social anxiety disorder, threat-related insula activity and its functional connectivity with the ACC are also altered (100). These findings suggest that ACC–insula interactions help integrate bodily signals with emotional salience and threat evaluation.

Rodent studies provide evidence that the rodent insula contributes to anxiety-like behavior and has bidirectional connections between the ACC and insula (101). When muscarinic cholinergic receptors are activated in the insula, rats showed decreased anxiety-like behavior in the elevated plus maze and inhibiting these receptors increased it (102). The effects of local insular activity also vary along the rostrocaudal axis. Increasing activity in rostral insular regions tends to increase anxiety-like behavior, whereas increasing activity in more caudal regions can reduce it (103). Thus, the insula is not functionally uniform, and its contribution to anxiety depends on the subregion and local circuit involved.

### ACC-BNST circuits

While a distinct brain structure, the BNST is considered part of the extended amygdala based on shared cytoarchitecture and strong interconnections with amygdalar and basal forebrain regions (104–106). The BNST is involved in sustained threat responding and anxiety-like states and complements amygdala-centered mechanisms that are more often emphasized for brief, cue-triggered defensive responses (104, 107, 108). Direct evidence for ACC–BNST connectivity in primates is limited but anatomically clear for the sgACC. In macaques, area 25 sends projections to the BNST, as well as to the amygdala, hypothalamus, periaqueductal gray, and other structures involved in affective and autonomic regulation (73). This pathway provides a route through which the sgACC may influence sustained threat and autonomic states.

Most mechanistic evidence comes from rodents and concerns the broader mPFC rather than the ACC specifically. In mice, chemogenetic inhibition of BNST-projecting mPFC neurons increases freezing to a partially reinforced threat cue but not to a consistently reinforced cue, indicating that this pathway limits defensive responding under conditions of threat uncertainty (109). Other mouse studies showed that BNST effects depend on subregion, cell type, and projection target. Activation of the oval BNST increased avoidance and respiratory responses, whereas activation of the anterodorsal BNST reduced these responses (110). In rats, pharmacological inactivation of the anterior BNST also blocks defensive behavior induced by alarm pheromone, further supporting its role in responses to diffuse or uncertain threat (111). Together, these rodent studies show that mPFC–BNST signaling can limit defensive responses to uncertain threat and that BNST regulation of anxiety-related behavior depends on subregion, neuronal identity, and projection target.

### An integrative model of ACC-centered anxiety circuits

**Figure 2:**
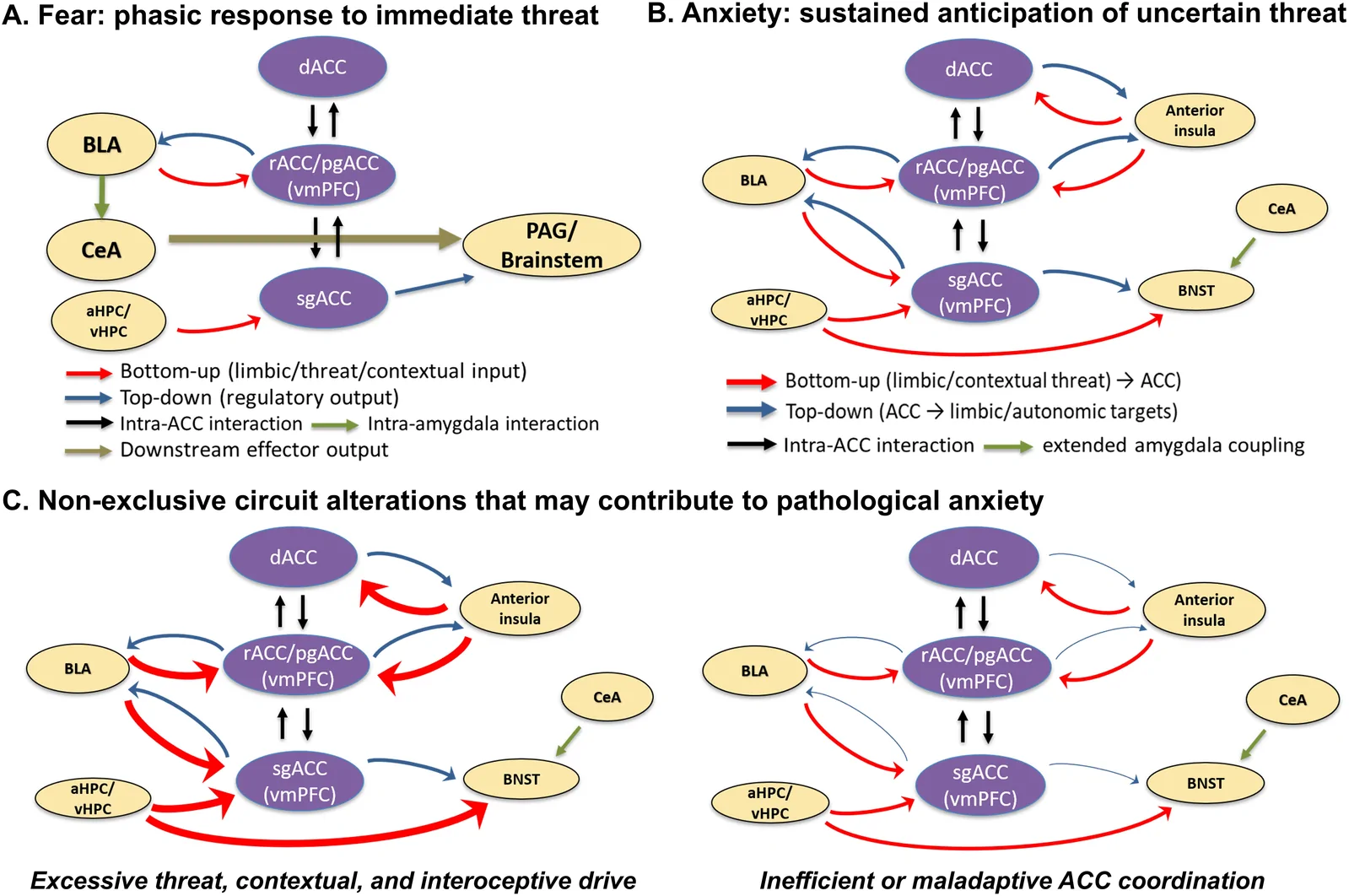
Working models of neural circuitry in fear and anxiety and conceptual modes of dysregulation. **(A)** Schematic representation of the core phasic fear network engaged by immediate or predictable threats. Limbic and contextual inputs from the basolateral amygdala (BLA) and hippocampus, together with top-down ACC modulation, influence amygdala, hypothalamic, periaqueductal gray, and brainstem systems that organize defensive and autonomic responses. **(B)** Schematic representation of the broader network engaged during sustained or uncertain threat, with ACC subregions serving as integrative nodes. The ACC receives threat-related, contextual, and interoceptive information from the BLA, anterior hippocampus in primates or ventral hippocampus in rodents (aHPC/vHPC), and anterior insula and modulates limbic and autonomic targets, including the bed nucleus of the stria terminalis (BNST). **(C)** Non-exclusive circuit alterations that may contribute to pathological anxiety. Pathological anxiety may involve relatively excessive bottom-up threat, contextual, or interoceptive drive and/or inefficient or maladaptive top-down ACC modulation of limbic and autonomic circuits. Arrows indicate the proposed direction of influence and do not necessarily represent direct monosynaptic connections or uniformly excitatory or inhibitory effects.

Fear and anxiety engage overlapping neural systems, but immediate and well-defined threats rely more heavily on amygdala-centered circuits that rapidly organize autonomic and defensive responses, whereas sustained and uncertain threats recruit broader cortical–limbic interactions involving the ACC, hippocampus, insula, and BNST (**Figures 2A, B**). Within this broader network, the ACC functions as an integrative hub that combines threat-related, contextual, interoceptive, and autonomic information and uses this information to shape behavioral responses. Inputs from the BLA convey information about learned threat and emotional significance, the anterior hippocampus provides contextual and memory-related information, and the anterior insula conveys information about internal bodily states. The BNST integrates cortical and subcortical inputs during sustained and uncertain threat. Through reciprocal connections with these regions and outputs to autonomic and defensive systems, the ACC can adjust vigilance, threat evaluation, and behavioral responses according to the current context (58, 59, 71–81, 85, 90, 91, 93, 95–103, 109–112).

A major function of the ACC-centered network is top-down modulation of limbic and defensive circuits. However, this modulation is not uniformly inhibitory. Its effect depends on the ACC subregion, projection target, and behavioral condition. Among ACC subregions, the dACC supports salience and conflict monitoring, sustained threat anticipation and action selection, whereas pgACC and sgACC regions contribute emotional appraisal, threat–safety discrimination, and context-dependent modulation of limbic activity. The sgACC, particularly area 25, integrates affective, motivational, and autonomic information and can either constrain or facilitate threat responding through its projections to the amygdala, BNST, hypothalamus, periaqueductal gray, and related structures. Thus, top-down ACC output may suppress, enhance, or reorganize anxiety-related responses rather than simply inhibit them.

Adaptive anxiety depends on coordinated interactions between bottom-up inputs and ACC-mediated modulation. Threat, contextual, and interoceptive signals must be evaluated accurately, and the resulting response must be matched to the intensity, predictability, and duration of the threat. Pathological anxiety may arise when this balance is disrupted by excessive or poorly contextualized bottom-up input, impaired threat–safety discrimination, or inefficient or maladaptive ACC modulation of limbic and autonomic circuits (**Figure 2C**). These disturbances may produce hypervigilance, threat generalization, sustained autonomic arousal, avoidance, and pathological worry. The mechanisms shown in **Figure 2C** should therefore be viewed as overlapping rather than mutually exclusive. Excessive amygdala, hippocampal, insular, or BNST drive can occur together with altered ACC regulation, and the relative contribution of each mechanism may differ across individuals and disorders. PTSD, for example, may involve both heightened threat-related input and deficient context-dependent regulation, whereas GAD may involve persistent uncertainty processing, heightened interoceptive and anticipatory signaling, and inefficient modulation of extended threat circuits. This model places the ACC at the center of anxiety regulation while recognizing that its role is integrative and pathway-dependent rather than exclusively inhibitory.

## ACC alterations in anxiety-related disorders and neurodegenerative diseases

The ACC is an integrative hub that links cognitive and emotional processing with autonomic and behavioral responses (19–21). In humans and non-human primates, the ACC is involved in motivation, decision making, learning, cost-benefit calculation, conflict and error monitoring, social behavior, apathy, attention, dysregulation of autonomic functions, anxiety, and emotional instability (19–22, 113–115). Lesions involving areas 24 and 32 in macaques impair social interaction, whereas lesions of the anterior cingulate gyrus reduce the valuation of social information (113, 115). As a treatment for severe forms of these disorders, cingulotomy procedures targeting ACC regions have helped some patients by decreasing feelings of distress and stabilizing emotions (19, 114). These findings illustrate the broad contribution of the ACC to affective and social behavior. Consistently, structural, functional, connectivity, and neurochemical alterations of the ACC have been reported in generalized anxiety disorder (GAD), social anxiety disorder (SAD), panic disorder, obsessive-compulsive disorder (OCD), and post-traumatic stress disorder (PTSD) (**Table 3**). However, these alterations are not uniform in direction or location. Increased and decreased ACC activity have both been reported, depending on the ACC subregion, experimental task, symptom dimension, medication status, and stage of illness (116–119). The following sections summarize convergent and divergent ACC findings across these disorders and in neurodegenerative diseases in which anxiety is a prominent clinical symptom.

**Table 3:** Changes in the ACC in anxiety disorders and diseases with anxiety as a symptom.

Disorder	ACC Alterations (as described in text)	Key Refs
Generalized Anxiety Disorder (GAD)	• ACC–amygdala circuitry dysfunction • ↓ frontolimbic structural integrity, altered rACC-limbic connectivity (amygdala, hippocampus) • Failure to recruit pgACC activity and negative pgACC–amygdala coupling during emotional conflict	(120–123)
Social Anxiety Disorder (SAD)	• ↑ rACC activation during facial processing • ↓ right insula-dACC connectivity during threat processing • ↑ ACC glutamate (MRS), associated with clinical impairment	(100, 124–127)
Panic Disorder	• Altered connectivity between ACC and limbic/salience regions (amygdala, insula, hippocampal formation) • ↓ gray matter volume in right dorsal/rostral ACC • ↑ fractional anisotropy in the left anterior cingulum, associated with symptom severity • Task-dependent ACC findings: reports of ACC-amygdala circuit hyperactivity, but also reduced ACC responses to emotional faces	(128–132)
Obsessive-Compulsive Disorder (OCD)	• dACC hyperactivity during symptom provocation and error processing (-exaggerated error and conflict monitoring) • ↑ GABA levels • ↑ GABA: Glutamate ratio • ↓ glutamate-glutamine (Glx) (MRS) • FDG-PET: ACC hypermetabolism, with a shift toward hypometabolism with progression	(116, 133–138)
Post-Traumatic Stress Disorder (PTSD)	• ↓ ACC volume in chronic PTSD • ↓ vmPFC and rostral/ventral ACC recruitment during emotional processing and symptom provocation • ↑ ACC glutamate–glutamine/creatine ratio in pediatric PTSD • ↓ left ACC white-matter connectivity • Altered functional connectivity: ACC-right hippocampus, ACC-left cerebellum • Altered ACC–amygdala regulation	(139–150)
Alzheimer’s Disease (AD)	• ↑ ACC perfusion (hyperperfusion) associated with anxiety severity • Associated with cerebral β-amyloid burden; ACC-specific relationship uncertain. • ACC dysfunction linked to broader neuropsychiatric symptoms	(151–154)
Parkinson’s Disease (PD)	• ↓ bilateral ACC volume • Serotonergic disruption in ACC linked to anxiety severity • Exploratory PET: anxiety severity related to reduced dopamine/norepinephrine transporter binding in ACC and other limbic regions	(155–158)

GAD is characterized by persistent and overwhelming episodes of worry that are difficult to control (2). Neuroimaging studies indicate altered ACC–amygdala interactions during emotional processing and regulation. During an emotional-conflict task, patients with GAD failed to recruit the pgACC and did not show the negative pgACC–amygdala coupling observed in healthy participants (120). Other studies have identified altered amygdala-centered functional connectivity and reduced structural integrity of frontolimbic pathways involving ventral prefrontal and ACC regions (121, 122). Altered anticipatory activity in the ACC and amygdala has also been reported in GAD and may vary with symptom severity and treatment response (123). Together, these findings indicate impaired recruitment of ACC-centered regulatory processes, although the direction and anatomical distribution of the abnormalities depend on the task and patient population.

SAD is mainly characterized by intense fear and avoidance of social situations in which an individual may be observed or evaluated by others (124). fMRI studies have reported heightened rACC responses during the processing of negative facial expressions, with ACC activity also varying with the severity of social-anxiety symptoms (125, 126). In addition, right anterior insula–dACC connectivity is reduced during threat processing in SAD (100). Magnetic resonance spectroscopy studies have reported elevated ACC glutamate concentrations that correlate with clinical impairment (127). These findings implicate altered interactions between ACC-mediated appraisal and salience-processing systems in social anxiety.

Panic disorder is characterized by recurrent, unexpected panic attacks and persistent anxiety about subsequent attacks (11). Neuroimaging studies have reported altered connectivity between the ACC and limbic or salience-network regions, including the amygdala, insula, and hippocampal formation (128). Structural MRI studies have identified reduced gray-matter volume in the right dorsal and rostral ACC (129, 130). Diffusion-tensor imaging has also shown increased fractional anisotropy in the left anterior cingulum, with greater values associated with greater symptom severity (131). Functional findings are less consistent. Altered ACC-amygdala activity and reduced cingulate responses during fearful-face processing have both been reported, suggesting that ACC dysfunction in panic disorder is task- and subregion-dependent (128, 132).

OCD is characterized by intrusive thoughts, urges, or images and repetitive behaviors or mental acts (133). Hyperactivity of the ACC, particularly the dACC, has been reported during symptom provocation and error processing and may contribute to exaggerated monitoring of errors, conflict, and the perceived need for corrective behavior (116, 134, 135). Neurochemical findings also vary across age groups and study designs. An MRS study of adults with OCD found elevated ACC GABA levels and a higher GABA-to-glutamate ratio without a significant difference in glutamate alone (136), whereas reduced ACC glutamatergic concentrations have been reported in pediatric OCD (137). A combined PET and fMRI study further suggested that ACC metabolic activity may vary with disease progression (138).

PTSD is characterized by intrusive symptoms, avoidance, negative alterations in cognition and mood, and heightened arousal following trauma exposure (133). Structural studies have reported reduced ACC volume in chronic PTSD, with ACC morphology also associated with psychophysiological responses to threat (139–141). Functionally, PTSD has been conceptualized as involving altered ACC regulation of amygdala and other threat-processing systems (142). Task-based studies and meta-analyses have frequently identified reduced recruitment of the vmPFC and rostral or ventral ACC during emotional processing and symptom provocation (143–146). However, increased activity in midcingulate or dorsal ACC regions has also been reported, particularly during threat detection and salience processing (143). Greater prefrontal and ACC recruitment during inhibitory-control tasks has been associated with better fear inhibition in trauma-exposed individuals (147). Additional findings include reduced white-matter integrity in the left anterior cingulum (148), increased ACC glutamate–glutamine relative to creatine in pediatric PTSD (149), and altered dACC functional connectivity with hippocampal and cerebellar regions (150). These findings indicate that PTSD involves heterogeneous ACC alterations rather than a uniform reduction in ACC activity.

Anxiety is also a common symptom of many neurodegenerative diseases, including AD and PD. In mild AD, anxiety severity has been associated with hyperperfusion of the bilateral ACC (151). Anxiety and other neuropsychiatric symptoms have also been associated with cerebral amyloid burden, although the anatomical distribution and causal significance of this relationship remain uncertain (152–154). More broadly, ACC and salience-network dysfunction have been implicated across several neuropsychiatric symptoms in AD (152, 153). In PD, anxiety has been associated with reduced gray-matter volume in the ACC and with alterations in broader fear and limbic cortico-striatal circuits (155, 156). In addition, serotonergic degeneration in the sgACC and dACC has been associated with the severity of anxiety, depression, and apathy in patients with early PD (157). This severity is also linked to loss of dopaminergic and noradrenergic signaling between the ACC and limbic brain regions, including the amygdala, as shown in exploratory PET analyses (158). These findings support ACC involvement in anxiety-related symptoms in PD but do not establish whether ACC abnormalities are primary drivers of anxiety or consequences of more widespread neurodegeneration. Across conditions, ACC abnormalities reflect partly shared but distinct processes. pgACC/sgACC changes are more often linked to emotional appraisal, contextual regulation, and autonomic responses, whereas dACC/aMCC alterations involve salience, conflict monitoring, and sustained threat. GAD and SAD primarily affect frontolimbic and salience-network regulation, OCD emphasizes excessive performance monitoring, and PTSD combines reduced ventral regulation with increased dorsal threat activity. In AD and PD, these changes occur within broader neurodegenerative pathology. Thus, similar ACC imaging abnormalities may reflect different mechanisms across disorders.

Although resting-state and task-based imaging provide important evidence for ACC alterations across anxiety-related conditions, they do not establish the direction or causal function of the observed relationships. Findings may also be influenced by differences in task design, symptom heterogeneity, comorbidities, medication exposure, and analytic procedures (118, 119). Human imaging should therefore be used to identify candidate subregions and circuits for mechanistic investigation rather than to infer direct causal control. Projection- and cell type-specific experiments in animals can test these hypotheses, whereas human and non-human-primate imaging can determine whether the resulting circuit models are relevant to clinical symptoms and large-scale network organization.

## Conclusions and perspective

The ACC is a major hub for cognitive, emotional, and autonomic functions through the intrinsic properties of its neurons and their local and long-range connections with other brain regions. It integrates information from the external environment, internal bodily states, memory, and ongoing goals to generate adaptive responses that promote survival. The ACC is a key component of multiple circuits involved in anxiety regulation, including those connecting it with the amygdala, insula, hippocampus, and BNST. These circuits contribute to threat evaluation, contextual processing, interoception, autonomic regulation, and social behavior. Adaptive anxiety depends on the ability to distinguish safety from threat and to adjust behavioral and physiological responses according to the intensity, predictability, and duration of potential danger. Although many of these circuit features are broadly conserved across mammals, the contribution of the ACC varies across subregions, projection pathways, and behavioral contexts. A deeper understanding of ACC function is therefore essential for defining how adaptive anxiety becomes pathological and for identifying more precise therapeutic interventions.

Although the ACC and its connected regions have been extensively studied in anxiety, the cellular, molecular, and epigenetic mechanisms that regulate these circuits remain poorly understood. Only a small number of molecular factors have been examined directly in the ACC. For example, *PPP1R9A*/neurabin regulates anxiety-like behavior through the ACC, whereas *COMT* and *CRFR1* have been linked to anxiety more broadly but have not been established as ACC-specific regulators (64, 159, 160). Transcriptomic studies of the human sgACC have identified cross-diagnostic and disorder-specific gene-expression changes in major psychiatric disorders (161), and studies in mice have reported molecular changes associated with aging and anxiety-like behavior (162). Epigenetic regulation may also contribute, as histone deacetylase-dependent mechanisms within the cingulate cortex can alter innate anxiety-like behavior (163). Other epigenetic mechanisms, such as DNA methylation, enhancer regulation, and chromatin remodeling, remain insufficiently explored. Future studies combining single-nucleus RNA sequencing, spatial transcriptomics, ATAC-seq, and other epigenomic approaches with tract tracing, electrophysiology, calcium imaging, and projection-specific manipulation will be important for identifying the genes and cell populations that regulate defined ACC circuits.

Although ACC dysfunction is well established in anxiety disorders and other neuropsychiatric diseases with anxiety as a clinical presentation, such as AD and PD, the shared and disease-specific mechanisms of anxiety-related pathology involving ACC in these conditions are not fully understood. Similar changes in ACC activity or connectivity may reflect common features such as hypervigilance, impaired safety learning, autonomic hyperarousal, or persistent negative affect, whereas other alterations may result from disease-specific pathology. Longitudinal imaging and molecular studies will be needed to determine whether ACC abnormalities precede anxiety symptoms, emerge as a consequence of chronic disease, or change with treatment. Machine-learning approaches may help identify reproducible circuit patterns and predict treatment responses, but these models will require large, well-characterized datasets and independent validation.

Rodent models remain essential for causal analysis of ACC genes, cell types, and projection pathways. They also permit longitudinal studies across development, adulthood, aging, and disease progression. However, rodent medial frontal regions do not correspond directly to all primate ACC subregions, and behavioral tests such as the open field, light–dark box, and elevated plus maze measure specific dimensions of avoidance, exploration, and arousal rather than complete human anxiety disorders. Cross-species interpretation will therefore be strongest when studies clearly define the cytoarchitectonic region, cell population, and pathway being manipulated and combine several behavioral and physiological measures. Non-human primate studies provide an important bridge between mechanistic rodent experiments and human imaging findings.

Growing evidence suggests that the ACC may provide a promising target, with several existing interventions already showing effects on ACC function. For example, in a mouse model of chronic pain-induced anxiety, systemic duloxetine reduced pain and anxiety-like behavior and was accompanied by altered serotonergic and glutamatergic signaling in the ACC, although these findings did not establish that ACC changes directly mediated the anxiolytic effect (164). In addition, non-pharmacological approaches such as meditation and cognitive behavioral therapy have been found to modulate ACC activity and improve anxiety outcomes, likely through broader changes in cognitive-control, salience, and affective networks (165, 166). Given the complex involvement of ACC in anxiety, its activity should not be viewed as uniformly beneficial or harmful. Future treatments should aim to normalize specific maladaptive circuit states while preserving the adaptive functions of anxiety.

Our understanding of ACC participation in anxiety circuits is still evolving. The major next step is to connect the structural and functional abnormalities identified in human studies with causal molecular and cellular mechanisms in defined ACC pathways. Integrating human imaging and clinical phenotyping with primate tracing and causal circuit studies in rodents will help determine how ACC networks function under normal conditions, become disrupted in disease, and respond to treatment. Such cross-species and multiscale approaches should provide a more precise understanding of how adaptive anxiety becomes pathological and support the development of interventions directed at specific ACC-centered circuit mechanisms.
